# Criteria for early diagnosis of mandibular third molar agenesis based on the developmental stages of mandibular canine, first and second premolars, and second molar: a retrospective cohort study

**DOI:** 10.1186/s12903-023-03349-5

**Published:** 2023-09-08

**Authors:** Hyuntae Kim, Hong-Keun Hyun, Teo Jeon Shin, Young-Jae Kim, Jung-Wook Kim, Ki-Taeg Jang, Ji-Soo Song

**Affiliations:** 1 Department of Pediatric Dentistry, Seoul National University Dental Hospital, 101 Daehak-ro, Jongno-gu, Seoul, 03080 Republic of Korea; 2 Department of Pediatric Dentistry, Dental Research Institute, School of Dentistry, Seoul National University, 101 Daehak-ro, Jongno-gu, Seoul, 03080 Republic of Korea

**Keywords:** Diagnostic criteria, Tooth agenesis, Mandibular third molar, Dental developmental stage, Survival analysis

## Abstract

**Background:**

Permanent first molars with severe dental caries, developmental defects, or involved in oral pathologies are at risk of poor prognosis in children. Accordingly, using the third molar to replace the first molar can be a good treatment option when third molar agenesis is predicted early. Thus, this retrospective cohort study aimed to develop criteria for early detection of mandibular third molar (L8) agenesis based on the developmental stages of mandibular canine (L3), first premolar (L4), second premolar (L5), and second molar (L7).

**Method:**

Overall, 1,044 and 919 panoramic radiographs of 343 males and 317 females, respectively, taken between the ages of 6 and 12 years were included. All developmental stages of L3, L4, L5, L7, and L8 were analyzed based on the dental age, as suggested by Demirjian et al. The independent t-test was used to assess age differences between males and females. The rank correlation coefficients were examined using Kendall’s tau with bootstrap analysis and Bonferroni’s correction to confirm the teeth showing developmental stages most similar to those of L8s. Finally, a survival analysis was performed to determine the criteria for the early diagnosis of mandibular third molar agenesis.

**Results:**

Some age differences were found in dental developmental stages between males and females. Correlation coefficients between all stages of L3, L4, L5, and L7 and L8 were high. In particular, the correlation coefficient between L7 and L8 was the highest, whereas that between L3 and L8 was the lowest.

**Conclusion:**

If at least two of the following criteria (F stage of L3, F stage of L4, F stage of L5, and E stage of L7) are met in the absence of L8 crypt, agenesis of L8 can be confirmed.

## Background

Tooth development is a very sophisticated and systematic process regulated by more than 300 genes and environmental factors [1]. However, abnormalities in tooth development may occur due to genetic or environmental issues during tooth development [2]. The first molar is usually the first permanent tooth to start developing [3], and it erupts into the oral cavity at approximately 6 years of age [4]. This tooth exhibits a low degree of calcification immediately after eruption. And it is covered by a gingival plate and its vertical occlusal relationship remains unestablished for a long period. Thus, it is vulnerable to severe dental caries [5], possibly leading to its loss at a young age.

Notably, hard tissue deposition on the first molar begins around the time of birth. If there are some negative factors influencing tooth development during this time, the first molar can have developmental defects such as molar incisor hypomineralization (MIH) and molar root incisor malformation (MRIM). MIH is a relatively common condition and is characterized by defective enamel of the permanent incisors and first molars, with an incidence of 4-25%. Teeth affected by this condition have demarcated opacities of a different color, and owing to the soft and porous enamel of these teeth, they are prone to posteruptive breakdown and dental caries [6]. Therefore, early treatment is required for the affected first molars. However, MIH is often detected after extensive tooth destruction because patients with MIH are sensitive to stimuli, making it difficult to achieve adequate depth of local anesthesia and thereby leading to failure of behavioral control. Meanwhile, MRIM has only been identified relatively recently, and its prevalence remains unknown. The crowns of the first molars affected by MRIM are usually normal, but the roots are dysplastic, with narrowing pulp canals. Further, although the teeth affected by MRIM are susceptible to pulpal and periodontal disease, endodontic and periodontal treatment may be ineffective in severe cases [7]. Both MIH and MRIM are believed to be associated with systemic problems occurring from late pregnancy to early parturition; however, their etiologies are remains unknown.

Previous studies have reported that oral and maxillofacial pathologies occurred in approximately 10% of children and adolescents, and among these pathologies, odontogenic tumors and cysts had a prevalence of 5% and 10%, respectively [8]. The most commonly affected site by these pathologies was the mandible, followed by the maxilla, and they occurred more commonly in the posterior region than in the anterior region [9]. Further, these pathologies are known to cause resorption, displacement or mobility of the affected teeth and malocclusion; thus, their surgical removal is required despite the fact that most of them are benign. During surgery, a considerable number of affected teeth were extracted together [10, 11].

In contrast, the third molar is the last tooth to develop. According to previous studies, its development begins around the age of 9 years, and root growth is completed around the age of 20 years [12]. Notably, it is the only tooth that continues to grow beyond puberty. Given that the accuracy of sexual and skeletal maturity indices decreases with age [12], developmental stages of the third molar are widely used in forensic odontology for dental age estimation in adolescents [13, 14]. Furthermore, the third molar is the most commonly impacted tooth, and occasionally, it is necessary to extract this tooth because of follicular space enlargement, pericoronitis, and severe dental caries [15]. However, if the first molars with severe dental caries or developmental defects does not respond to palliative treatment, the long-term prognosis of the teeth remains uncertain, and extraction of affected teeth may be required. Moreover, in cases of oral pathologies such as odontogenic cysts or tumors, first molars can be affected by the pathology or need to be extracted during the procedure. In such cases, using the third molar to replace the missing first molar can be a good treatment option [16, 17]. In addition, extraction of the first molar facilitates the mesial drift of the second permanent molar. Further, more mesial movement of the second permanent molars can be expected if hopeless first molars are extracted in a timely manner [18]. Accordingly, it is especially noteworthy that early detection of third molar development or agenesis is beneficial for comprehensive orthodontic treatment planning.

Owing to the wide variability in third molar development, it is difficult to directly compare dental developmental stages with chronological age [19, 20]. Previous research, on the other hand, claims that the developmental stages of adjacent teeth are closely related to each other, and tooth agenesis can be predicted with high accuracy if the developmental stages of adjacent teeth are used together in the analysis [21, 22]. Further, canines, first and second premolars and second molars retain considerable developmental potency at the time of initiation of third molar development. Therefore, the goal of this study is to identify criteria for early diagnosis of mandibular third molar agenesis using the developmental stages of mandibular canine, first and second premolars, and second molars.

## Methods

### Ethical considerations

This retrospective cohort study was conducted with the approval of the Institutional Review Board of Seoul National University School of Dentistry, Seoul, Korea (IRB No: S-D20210002) and in accordance with the principles of the Declaration of Helsinki. The requirement for obtaining informed consent to use retrospective data was waived by the Institutional Review Board of Seoul National University School of Dentistry. The data of the present study were accessible only to H Kim (first author) and JS Song (corresponding author), and these data were stored in encrypted files by the research manager. The statistician performed statistical analysis using anonymous data.

### Sampling

A total of 9,715 Korean children and adolescents under the age of 20 who had undergone at least one digital panoramic radiographic examination for diagnosis and treatment from January 2020 to December 2020 at Seoul National University Dental Hospital were screened. The inclusion criteria were as follows: patients who had at least two consecutive panoramic radiographs, one of which showed third molar development and the other did not, and those whose final radiographs revealed no missing teeth, including third molars. The exclusion criteria were as follows: any patient with dental pathology caused by genetic syndromes, maxillofacial deformities, lesions in the mandible, abnormal teeth angulation, or dental development disorders such as amelogenesis or dentinogenesis imperfecta and poor image quality on panoramic radiographs. Panoramic radiographs were included if they met the criteria regardless of when they were taken. Further, given that third molar development was not observed before the age of 6 years, and most root developments were completed by the age of 12 years in the present study, panoramic radiographs taken between the ages of 6 and 12 were included in the study. All panoramic radiographs were acquired using OP-100® (Instrumentarium Dental, Tuusula, Finland) and Rayscan α-P® (Ray, Gyeonggi, Korea).

### Dental age assessment

Developmental stages of mandibular canine (L3), mandibular first premolar (L4), mandibular second premolar (L5), mandibular second molar (L7), and mandibular third molar (L8) were analyzed on panoramic radiographs based on the dental age, as suggested by Demirjian et al. [23]. They classified developing teeth into eight stages based on the degree of calcification, from stage A (during which the calcification begins at the superior level of the crypt) to stage H (during which the apex of the root is completely closed and the width of periodontal ligament space is uniform). In addition, stage “−1” was defined by no sign of follicle formation, and stage “0” was defined by the presence of only bony crypts without calcification. All abbreviations are shown in Table 1.

**Table 1:** List of abbreviations

Abbreviations	Full name
L3	Mandibular canine
L4	Mandibular first premolar
L5	Mandibular second premolar
L7	Mandibular second molar
L8	Mandibular third molar
ρ3	Correlation coefficients between L8 and L3
ρ4	Correlation coefficients between L8 and L4
ρ5	Correlation coefficients between L8 and L5
ρ7	Correlation coefficients between L8 and L7

The developmental stages were evaluated by two independent pediatric dental specialists: H Kim and JS Song. Intra- and interobserver agreements were calculated using 100 randomly selected panoramic radiographs. In cases of disagreements between the two observers, a consensus was reached by discussion. The mean age at stages 0 and A of L8, where L8 began to develop and appear on panoramic radiographs, were investigated. And the age distribution according to developmental stages of L3, L4, L5, and L7 in males and females was identified.

### Statistical analysis

The intra- and interobserver agreements were evaluated using Cohen’s weighted kappa. The Wilcoxon signed-rank test was used to compare the developmental stages between the left and right teeth, and the independent t-test was used to assess age differences between males and females according to the dental developmental stage.

To identify which teeth among L3, L4, L5, and L7 could be included in the subsequent survival analysis, the correlation between each tooth L3, L4, L5, and L7 and L8 was confirmed using Kendall’s tau, based on the order of the developmental stages. To evaluate differences between each correlation coefficient and present confidence intervals, the bootstrap method and Bonferroni’s correction were used. The confidence interval was estimated using population sampling, which was repeated 1,000 times.

Finally, the survival analysis was performed to establish criteria for diagnosing the development of L8 [21, 22]. Xt was defined by the developmental stage of each tooth (L3, L4, L5, and L7) identified at the particular time t, and Y_t_ was defined by the developmental stage of L8 identified at the particular time t. The value of X_t_ when each tooth (L3, L4, L5, and L7) developed the most while L8 did not reach stage 0 was defined as follows.

$$Z:= \underset{t\in \left\{t:{Y}_{t}=-1\right\}}{\text{max}}{X}_{t}$$

Consequently, according to the abovementioned definition of Z, the relation (X_t_, Y_t_) = (x, − 1) ⇒ Z ≥ x was established. Given that Z is a value between the time when L8 was not observed at all and the time when L8 began to be observed, it can be considered an interval-censored value. P (Z ≥ x) can be nonparametrically estimated using the ic-np function of the icenReg package in the R software. In this study, P (Z ≥ x) was set as a threshold value, and when each tooth’s developmental stage precedes this threshold, we considered that L8 had already developed. Further, this univariate analysis was repeated for the teeth showing developmental stages similar to those of L8, and finally, the decision was made with majority to evaluate the agenesis of L8.

## Results

A final study sample included 660 patients, with 343 males (1,044 panoramic radiographs) and 317 females (919 panoramic radiographs) (Table 2). The average number of panoramic radiographs obtained per patient was 3.2 ± 1.0, and the average time between two radiographic examinations was 19.6 ± 12.9 months.

**Table 2:** The numbers of panoramic radiographs obtained between the ages of 6 and 12 years

Gender	Age (years)							Total
	6	7	8	9	10	11	12	
Male	98	151	182	209	203	125	76	1,044
Female	78	144	172	177	166	112	70	919

The Cohen’s weighted kappa values for intra- and interobserver reproducibility were all greater than 0.94 (p < 0.001). The number of panoramic radiographs obtained when the stage of L8 was 0 was 338 and 316 in males and females, respectively. At this time, the average age of males was 9.8 ± 1.6 years, whereas the average age of females was 9.9 ± 1.8 years, and L8 developed earlier in males than in females (p = 0.003). The number of panoramic radiographs showing stage A of L8 in males and females was 315 and 296, respectively. At this time, the average age of males and females were 10.2 ± 1.5 and 10.4 ± 1.7 years, respectively. Similarly, regarding L8, compared with females, males transitioned from stage 0 to A at a younger age (p = 0.029).

Because there were no differences between the left and right teeth in terms of the developmental stages (p = 0.75, 0.95, 0.81, and 0.54 for L3, L4, L5, and L7, respectively), they were analyzed together. The development of L3, L4, L5, and L7 was faster in females than in males at stage D, E, F, and G; stage E, F, and G; stage E and F; and stage D, E, and G, respectively (Table 3). Therefore, the analysis for diagnosing L8 agenesis was conducted in terms of sex.

**Table 3:** Age distribution according to dental developmental stage of mandibular canines, first and second premolars, and second molars

		C stage		D stage		E stage		F stage		G stage	
		n	Age (year)	n	Age (year)	n	Age (year)	n	Age (year)	n	Age (year)
Canine	Male			156	6.8 ± 0.6	515	7.8 ± 0.8	1007	9.7 ± 1.1	299	11.3 ± 0.9
	Female			58	6.5 ± 0.5	305	7.3 ± 0.7	769	8.9 ± 1.0	405	10.5 ± 0.9
	p-value				0.011*		< 0.0001*		< 0.0001*		< 0.0001*
First premolar	Male			236	6.9 ± 0.6	663	8.3 ± 0.9	727	9.9 ± 1.0	315	11.2 ± 0.9
	Female			149	6.8 ± 0.6	534	8.1 ± 0.9	638	9.6 ± 1.0	348	11.0 ± 0.9
	p-value				0.108		< 0.0001*		< 0.0001*		0.044*
Second premolar	Male	56	6.6 ± 0.6	367	7.3 ± 0.8	624	8.7 ± 1.0	787	10.3 ± 1.0	215	11.6 ± 0.8
	Female	36	6.7 ± 0.7	263	7.2 ± 0.8	568	8.5 ± 1.0	667	10.1 ± 1.0	255	11.5 ± 0.8
	p-value		0.462		0.199		0.001*		0.004*		0.182
Second molar	Male	228	7.0 ± 0.7	517	8.0 ± 0.9	619	9.5 ± 0.9	531	10.7 ± 1.0	183	11.8 ± 0.8
	Female	145	7.0 ± 0.6	469	7.9 ± 0.9	559	9.3 ± 1.0	467	10.7 ± 1.0	191	11.7 ± 0.7
	p-value		0.925		0.015*		0.021*		0.643		0.014*

All age values are mean ± standard deviation

Independent t-test; *:statistically significant (p < 0.05)

The stages from C to G represent dental developmental stages suggested by Demirjian et al

The data for canine and first premolar at C stage were not included because of insufficient sample sizes

The results of correlations using Kendall’s tau, bootstrap method, and Bonferroni’s correction are as follows. In both males and females, the correlation coefficient between L7 and L8 (ρ7) was the highest, followed by that between L5 and L8 (ρ5) and between L4 and L8 (ρ4); conversely, the correlation coefficient between L3 and L8 (ρ3) was the lowest (Table 4). The likelihood of the order of “ρ7 > ρ5 > ρ4 > ρ3” was identical in 1,000 bootstrap samples, and its possibility was very high (1.000 and 0.999 in males and females, respectively). However, because the differences in correlation coefficients were so small (Table 5), and the value of the correlation coefficient is not proportional to the explanatory power, all teeth were used together to diagnose L8 agenesis regardless of the value of the coefficient values.

**Table 4:** Correlation coefficients (confidence interval) between mandibular third molars and mandibular canines, first and second premolars, and second molars

	Male	Female
	Third molar	
Canine	0.654* (0.626–0.681)	0.612* (0.581–0.643)
First premolar	0.672* (0.645–0.699)	0.624* (0.592–0.655)
Second premolar	0.685* (0.659–0.711)	0.670* (0.641–0.699)
Second molar	0.729* (0.706–0.752)	0.698* (0.671–0.725)

Kendall’s Tau with the Bootstrap method and Bonferroni’s corrections

* statistically significant (P < 0.05)

**Table 5:** Differences (confidence interval) between the correlation coefficients

	Male	Female
ρ7-ρ3	0.075* (0.045–0.105)	0.087* (0.055–0.119)
ρ7-ρ4	0.056* (0.025–0.087)	0.073* (0.042–0.104)
ρ7-ρ5	0.044* (0.016–0.071)	0.029* (-0.001-0.059)
ρ5-ρ3	0.036* (0.006–0.065)	0.058* (0.027–0.088)
ρ5-ρ4	0.016* (-0.011-0.042)	0.044* (0.014–0.074)
ρ4-ρ3	0.018* (-0.007-0.043)	0.014* (-0.015-0.042)

Kendall’s Tau with the Bootstrap method and Bonferroni’s corrections

ρ3, ρ4, ρ5, and ρ7 represent the correlation coefficients between the mandibular third molar and mandibular canine, first and second premolar, and second molar, respectively

* statistically significant (P < 0.05)

**Fig. 1:**
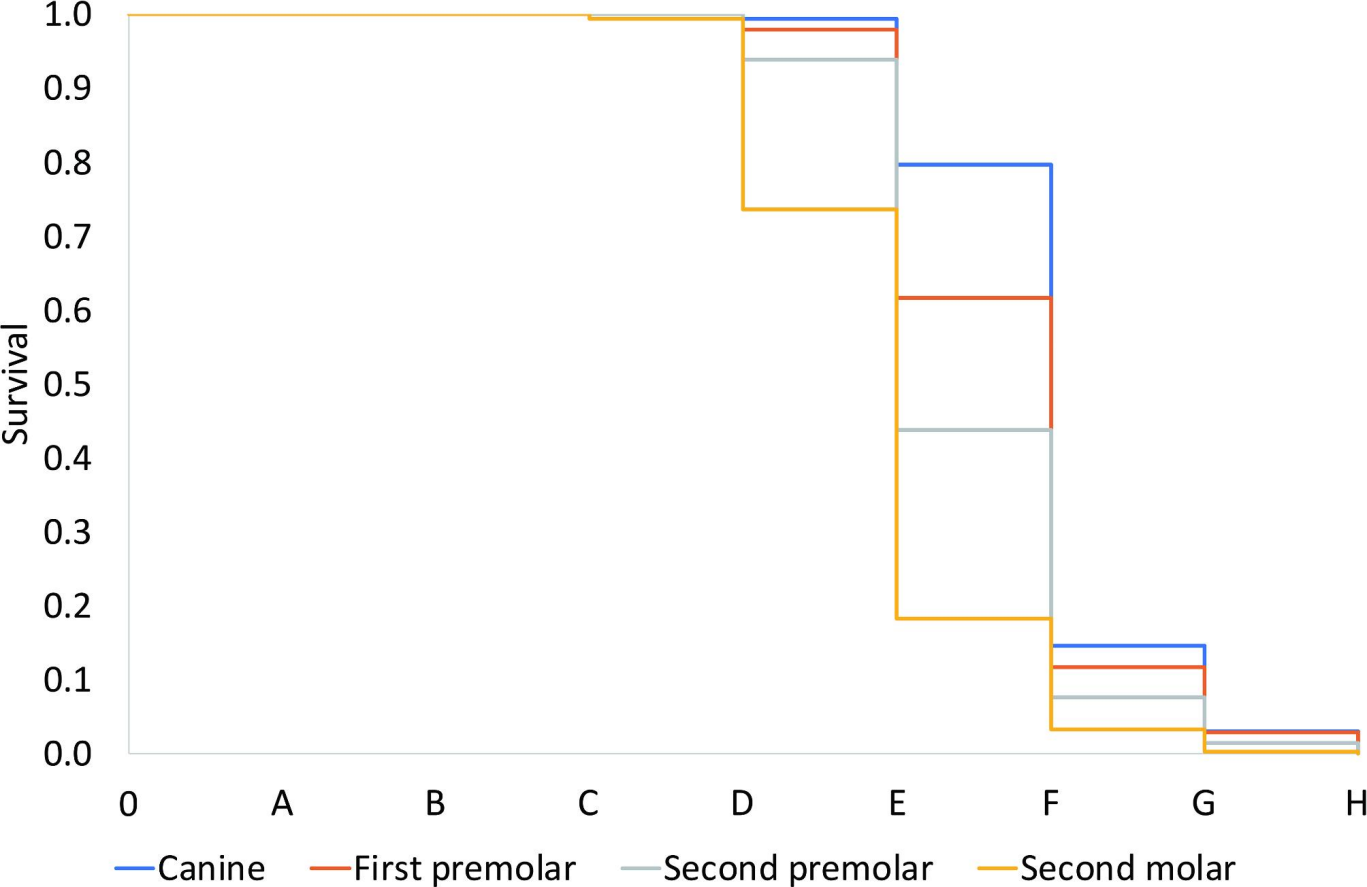
Results of estimates based on survival function according to the developmental stages of mandibular canines, first and second premolars, and second molars in the male. The number 0 represents the developmental stage when only bony crypts without any calcification are visible, and the letters from A to H indicate developmental stages suggested by Demirjian et al.

**Fig. 2:**
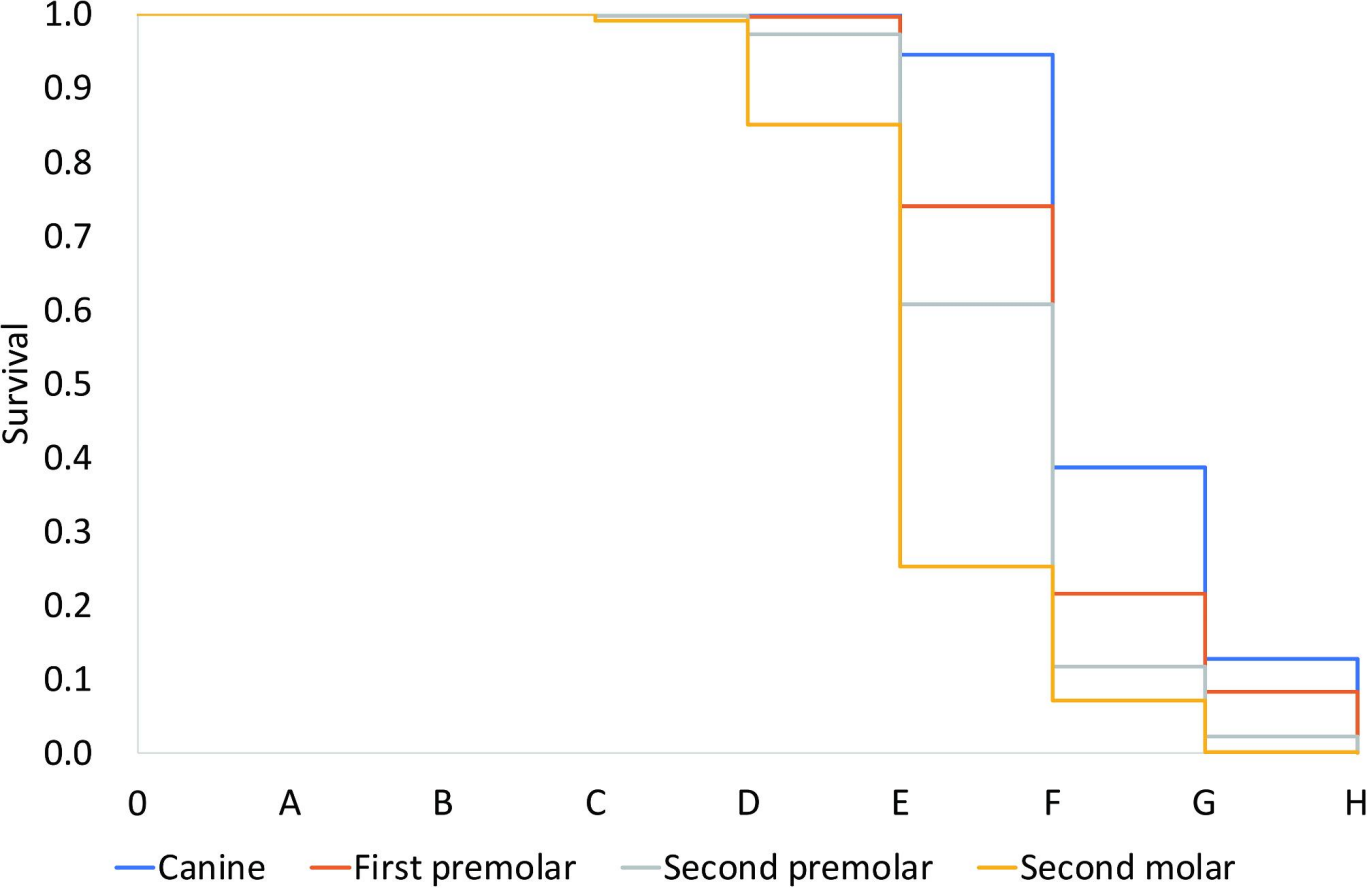
Results of estimates based on survival function according to the developmental stages of mandibular canines, first and second premolars, and second molars in the female. The number 0 represents the developmental stage when only bony crypts without any calcification are visible, and the letters from A to H indicate developmental stages suggested by Demirjian et al.

The results of the estimation using the survival function are shown in Table 6; Fig. 1, and Fig. 2. Stages in which the probability of L8 development decreased abruptly were as follows: Demirjian stage E in L3, L4, and L5 and Demirjian stage D in L7. As a result, agenesis of L8 can be confirmed if at least two of the following four criteria are met without L8 crypt: F stage of L3, F stage of L4, F stage of L5, and E stage of L7. The accuracy of these diagnostic criteria was 85.71% in males and 84.43% in females. The rate of false positives (L8 was not observed in actual data, but the presence of L8 was diagnosed according to the diagnostic criteria) were 6.53% in males and 11.71% in females, and false negatives (L8 was observed in actual data, but L8 was diagnosed as missing according to the diagnostic criteria) were 7.76% in males and 3.85% in females.

**Table 6:** Probabilities* of mandibular third molar development based on survival analysis

		Developmental stage							
		A	B	C	D	E	F	G	H
Canine	Male	1.000	1.000	1.000	0.994	0.797†	0.146	0.031	0.000
	Female	1.000	1.000	1.000	1.000	0.946†	0.387	0.128	0.000
First premolar	Male	1.000	1.000	1.000	0.980	0.617†	0.118	0.030	0.000
	Female	1.000	1.000	1.000	0.996	0.741†	0.216	0.084	0.000
s premolar	Male	1.000	1.000	1.000	0.939	0.438†	0.077	0.015	0.000
	Female	1.000	1.000	0.998	0.973	0.607†	0.118	0.023	0.000
s molar	Male	1.000	1.000	0.994	0.737†	0.183	0.034	0.003	0.000
	Female	1.000	1.000	0.992	0.851†	0.253	0.071	0.002	0.000

*Probabilities when each tooth of canine, first and second premolar, and second molar reached certain stage while third molar had just initiated its development

†Threshold at which the probability of mandibular third molar development abruptly decreased

## Discussion

The third molar provides unique information about tooth development because it is the only tooth whose entire developmental stages can be seen in panoramic radiographs [24]. However, when compared with other teeth in the permanent dentition, the development of the third molar has the greatest variation in terms of the morphology and timing of development, ranging from the age of 7 to 16 years [25]. Third molar impaction is a common dental problem caused by a lack of space in the retromolar area, ectopic eruption path, and shape of the mandible and third molars are usually removed surgically [15]. Meanwhile, permanent first molars are the most important teeth that play a key role in occlusion and mastication, so healthy survival of permanent first molars is critical from functional and developmental perspectives [26]. However, the first molars, which are expected to have a poor prognosis in early mixed dentition, can be extracted due to severe dental caries, developmental defects such as MIH and MRIM, and oral pathologies. In such cases, loss of the first molar may be followed by successful eruption of the second molar and, eventually, eruption of the third molar to complete the molar occlusion, though this is not always guaranteed [17]. Accordingly, if pediatric dentists and orthodontists can predict the agenesis of third molar development early, the best treatment plans, including first molar extraction, can be devised [27].

The mean ages at stage 0 of L8, when the crypt of L8 was first observed, were 9.8 ± 1.6 and 9.9 ± 1.8 years for males and females, respectively. When it came to stage A of L8, when calcification of the crown was first initiated, the mean ages for males and females were 10.2 ± 1.5 and 10.4 ± 1.7 years, respectively. These findings differ from those of other studies reporting that L8 development begins before the age of 9 years [28, 29]. Nonetheless, they are consistent with the results of a previous study on Korean population [30], suggesting that it may be due to racial differences. Concerning the beginning of L8 development, no observation was found before the age of 6 years in the present study. Meanwhile, L8s were first observed at the age of 6 years only in six males and four females, suggesting that L8 development begins later in the Korean population than in other racial groups.

Unlike first molars and incisors, which mature at a young age, the development of lateral segment teeth such as canines, premolars, and second molars was not completed during the early development of third molars. As a result, the development stages of L3, L4, L5, and L7 were analyzed in this study to evaluate L8 agenesis. And it revealed a strong correlation between L3, L4, L5, and L7 and L8. This can be attributed to the fact that these teeth begin to develop at a similar time and in a relatively late period [3], so all four teeth, L3, L4, L5, and L7 were used to evaluate L8 agenesis. Given this background, agenesis of L8 can be diagnosed if at least two of the following criteria are met without the presence of L8 on panoramic radiographs: F stage of L3, F stage of L4, F stage of L5, and E stage of L7. The accuracy of these diagnostic criteria was 85.71% for males and 84.43% for females. This rate was lower than that in previous studies that reported higher accuracy for predicting agenesis in the maxillary and mandibular second premolars [21, 22]. This difference could be due to the large variation in the developmental period of third molars between studies [24, 25].

Previous research supported that if the maxillary first molar is extracted before the eruption of the maxillary second molar, the maxillary second molar can erupt to the position of the missing first molar, forming an appropriate occlusion [31]. However, in the mandible, the mesial movement of the second molar may not be enough to create an ideal contact relation [32]. As a result, early extraction of the first molar is critical for achieving the ideal arrangement of the second molar. In general, it is recommended to extract the first molar around the age of 8–10 years, when the second molar is at Demirjian stage E or lower, where calcification of furcation area has just initiated [27]. In the present study, the average age of L8 at stage 0 was 9.8 years for males and 9.9 years for females. Furthermore, as shown in Table 3, the average ages of L3, L4, and L5 at F stage were over 9.7 years in males and over 8.9 years in females. The average ages of L7 at stage E were over 9.3 years. So, the presence of L8 can be confirmed after the age of 9 years. If the mandibular first molar is extracted around the age of 9 years, significant mesial movement of the mandibular second molar can be expected. As a result, the extraction of the first molar at an appropriate time facilitates the eruption of the second molar to the original position of the first molar, followed by the eruption of the third molar into the oral cavity by securing space for the eruption of the third molar; this will finally help to establish proper posterior occlusion [27, 33].

Even if the mandibular first molar is extracted early, L7 may exhibit a mesial inclination or L5 may exhibit a distal inclination, resulting in poor vertical occlusal contact. Further, if too much space remains in the lateral segment area, overbite may worsen due to the lingual tilting of the mandibular incisors [34]. Moreover, removal of the first molar can lead to skeletal changes such as mandibular asymmetry and rotation of the occlusal plane [35, 36]. In addition, it is important to consider the pre-existing malocclusion together. Based on these findings, if the first molar needs to be replaced by the third molar, comprehensive orthodontic treatment planning and the developmental stage of the second molar should be considered. In such cases, dentists should consider long-term orthodontic treatment if the first molar extraction space remains [34].

This study has several limitations. First, the study sample was limited to Korean children and adolescents; thus, it is difficult to generalize the findings of this study to other racial groups [29]. Second, although third molar agenesis is known to be common [37, 38], less than 5% of all patients screened in the present study exhibited L8 agenesis. Hence, it is hard to compare the developmental stages with L8 agenesis samples. The very low L8 agenesis was attributed to the fact that the frequency of third molar agenesis generally increases in patients with other tooth agenesis or genetic syndrome, who were excluded from this study [39]. Third, it has been reported that the rate of agenesis of the maxillary third molar is at least 1.5 times higher than that of the mandibular third molar [38, 40], but only the mandibular teeth were examined in this study. Lastly, the results of this study cannot be extrapolated to the patients with agenesis of teeth other than the third molars.

## Conclusions

This study reported some age differences between males and females in dental developmental stages. Correlation coefficients between all stages of L3, L4, L5, and L7 and L8 were high. ρ7 was the highest, followed by ρ5 and ρ4, and ρ4 was the lowest. Finally, L8 agenesis can be confirmed if at least two of the following criteria are met in the absence of the L8 crypt: F stage of L3, F stage of L4, F stage of L5, and E stage of L7 are met in the absence of the L8 crypt.

## Data Availability

The datasets analyzed during the current study are available from the corresponding author upon reasonable request.

## List of abbreviations

L3: Mandibular canine
L4: Mandibular first premolar
L5: Mandibular second premolar
L7: Mandibular second molar
L8: Mandibular third molar
ρ3: Correlation coefficients between L8 and L3
ρ4: Correlation coefficients between L8 and L4
ρ5: Correlation coefficients between L8 and L5
ρ7: Correlation coefficients between L8 and L7
